# Hepatic adrenal adenoma-rare tumor on right lobe of liver: a case report and literature review

**DOI:** 10.1186/s12893-020-00780-1

**Published:** 2020-06-10

**Authors:** Haibo Yu, Yadong He

**Affiliations:** Department of Hepatobiliary Surgery, Wenzhou Central Hospital, The Dingli Clinical Institute of Wenzhou Medical University, Wenzhou, Zhejiang, People’s Republic of China 325000

**Keywords:** Hepatic adrenal adenoma, Hepatic carcinoma, Hepatic adrenal ectopia

## Abstract

**Background:**

Hepatic adrenal ectopia is a common clinical diagnosis, whereas adrenal tumors developed from hepatic adrenal ectopia are rare. Hepatic adrenal tumors are easily misdiagnosed as hepatic carcinoma and frequently treated by unnecessary operations.

**Case presentation:**

A 50-year-old female patient was hospitalized due to B-ultrasonic detection of “right focal liver lesions.” After hospitalization, enhanced CT examination was performed. A 2.2 cm × 1.8 cm tumor was found in the seventh section of the right liver, as indicated by obvious enhancement of the arterial phase and low density during the portal vein and delay stages. Enhanced MRI examination detected a 2.0 cm × 1.8 cm tumor on the right liver, which was considered a “primary hepatic carcinoma”. The patient was treated by open hepatectomy and recovered well after the operation. The postoperative pathological diagnosis was hepatic adrenal adenoma. No relapse was observed through the 1-year follow-up visit.

**Conclusions:**

According to imaging manifestations, pathological immunohistochemical treatment, alpha fetoprotein (AFP) and clinical features, hepatic adrenal tumors should be considered in the diagnosis of hepatic carcinoma to prevent misdiagnosis. Hepatic adrenal tumors should be ruled out during the diagnosis to avoid unnecessary operation.

## Background

Heterotopic adrenal tissue is commonly diagnosed in the clinic. According to Vestfrid MA [1], heterotopic adrenal tissue is detected in 50% of newborns and children and in 1% of adults. Heterotopic adrenal tissue exists in many parts of the human body and is mostly common on retroperitoneal fats close to the adrenal gland. Ectopic adrenal tissue includes renal adrenal ectopia and hepatic adrenal ectopia. These ectopic adrenals, particularly hepatic adrenal ectopia, may occasionally cause hyperplasia or tumors. Adrenal tumors are often misdiagnosed as liver cancer because of their similar imaging manifestations [2].

In 1935, Weller first reported hepatic adrenal ectopia [3]. However, the first case of hepatic adrenal ectopia was detected through the autopsy of a 27-year-old female who died from phthisis in 1885. Weller believed that adrenal ectopia is caused by partial or complete integration of the adrenal glands with the kidney or liver. In 1968, Dolan detected five cases of adrenohepatic fusion (AHF) from 115 autopsies and categorized them as adrenohepatic adhesion (AHA) and AHF [4]. In 1976, Honoré LH reported AHA and AHF and analyzed their relevant mechanisms; the results showed that such tumors that develop from ectopic adrenals are rare and should be investigated by surgeons and pathologists to ensure systematic accumulation of many cases and reduce misdiagnosis [5]. In 1991, Honma systematically introduced AHF and defined it as the combination of liver tissues and (right) adrenal glands with tight mixing of their parenchyma cells. The pathological state of AHF differs from adrenohepatic adhesion. Honma detected 63 AHF cases from 636 autopsies, indicating that AHF was not rare [6].

This study reports a case of a patient with rare hepatic adrenal adenoma, which was misdiagnosed as liver cancer and treated by tumor excision.

## Case presentation

A female patient was hospitalized for “B-ultrasonic detection of focal liver lesion for 1 month.” The patient had neither history of hypertension, diabetes, hepatitis B, or hepatitis C nor symptoms of stomachache, abdominal distension, nausea, dizziness, or change in stool properties. The patient had no family history of genetic diseases or tumors and had not taken drugs for a long period.

**Fig. 1:**
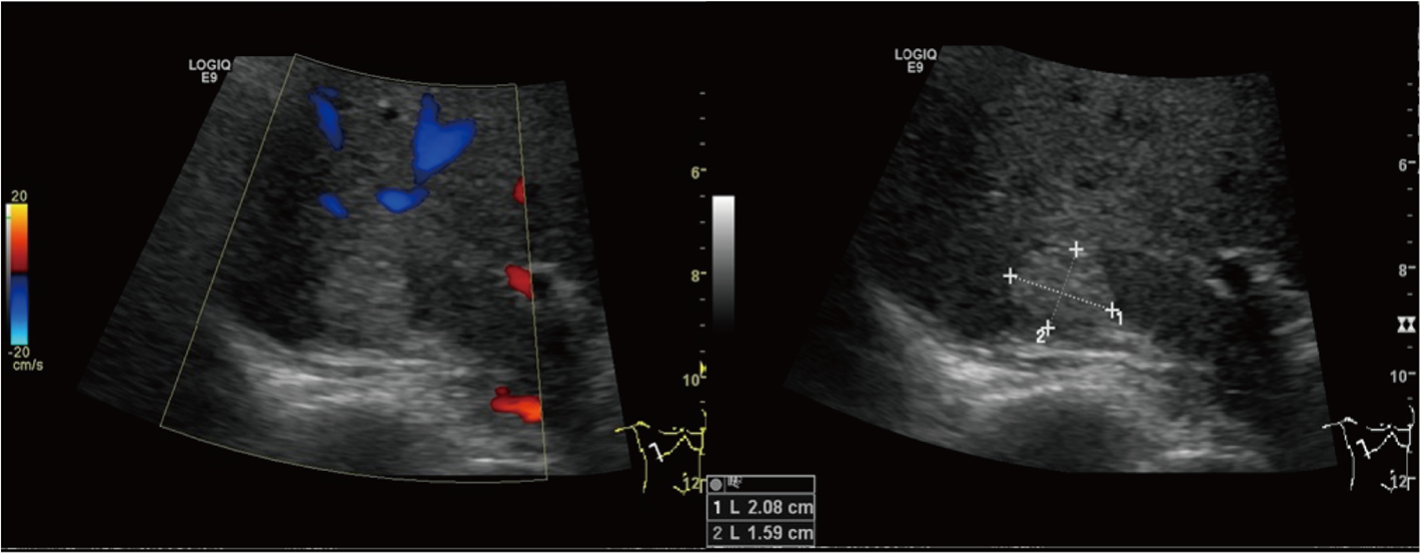
B-ultrasound reexamination showing a 2.1 cm × 1.6 cm echo node on the right lobe of the liver

**Fig. 2:**
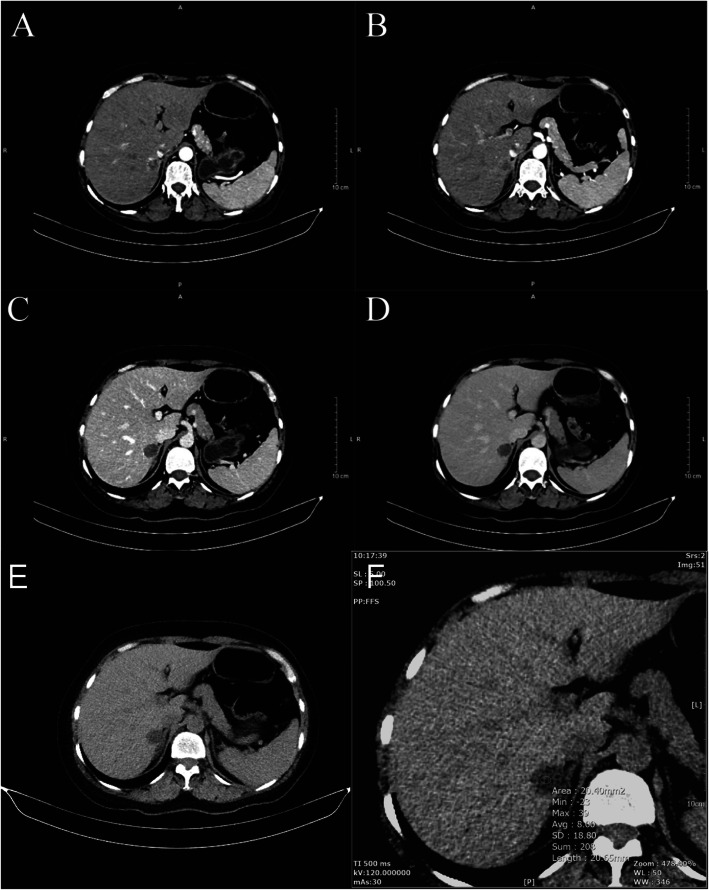
Enhanced CT of the abdomen showing an irregularly enhanced node at the seventh section of the arterial phase. The node contained edges enhanced in a circular manner and a few dense fat particles. The enhancement was evident during the venous portal and delayed stages. The focus size was 2.2 cm × 1.8 cm, showing clear boundaries. The average ordinary CT value of the tumor was 8 Hu

**Fig. 3:**
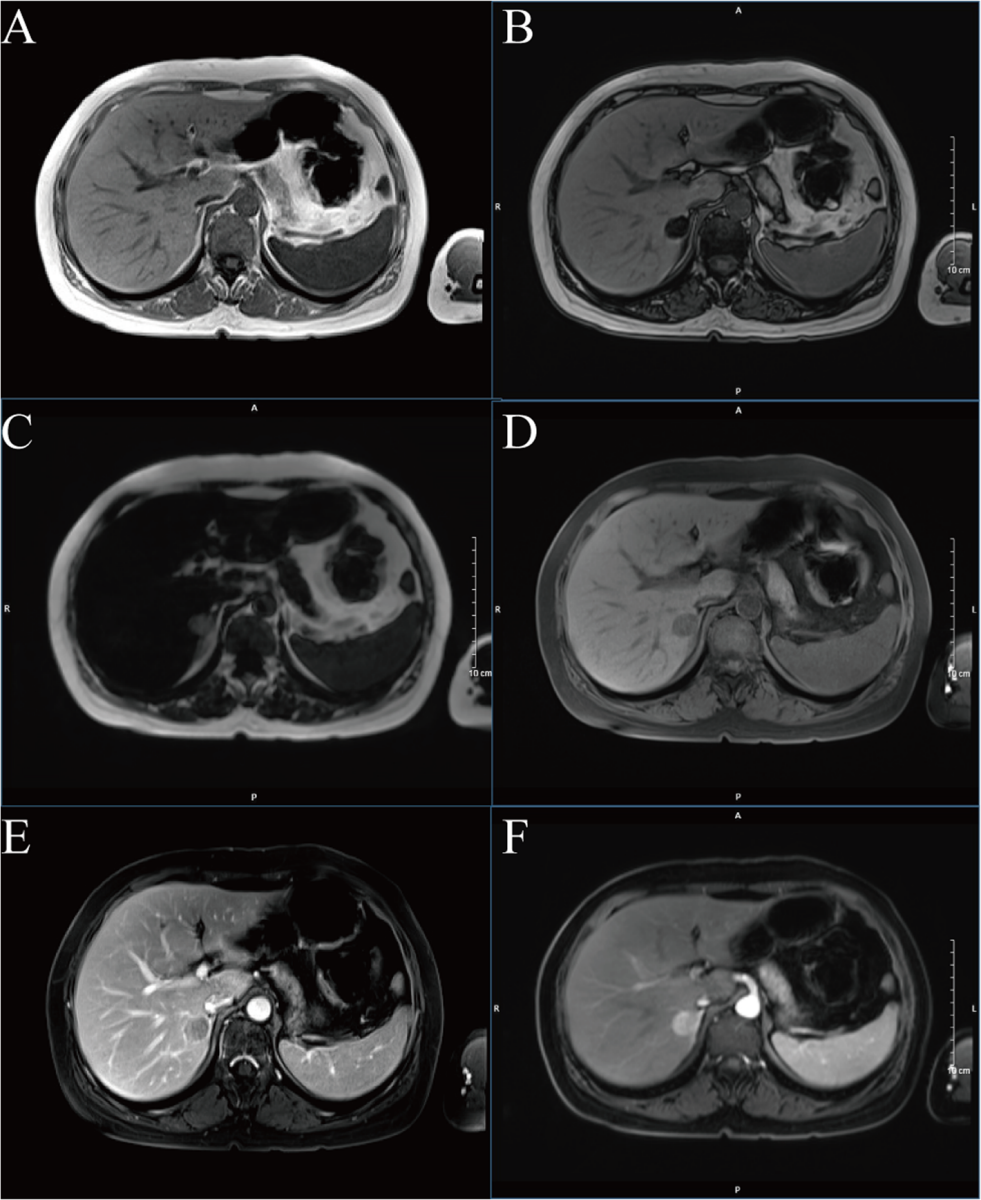
Enhanced magnetic resonance imaging (MRI) uncovered an abnormal signal of the nodule (20 mm × 18 mm with clear boundary) at the S7 section of the liver. T1WI presented equisignals, and the antiphase was considerably lower than the in-phase. T2WI and T2WI + FS presented slightly higher signals, DWI presented a high signal, and the ADC presented a slightly lower signal. These signals were considerably enhanced during enhanced arterial phase scanning but disappeared during the venous portal and delayed stages. All signals detected were located lower than the liver parenchyma

**Fig. 4:**
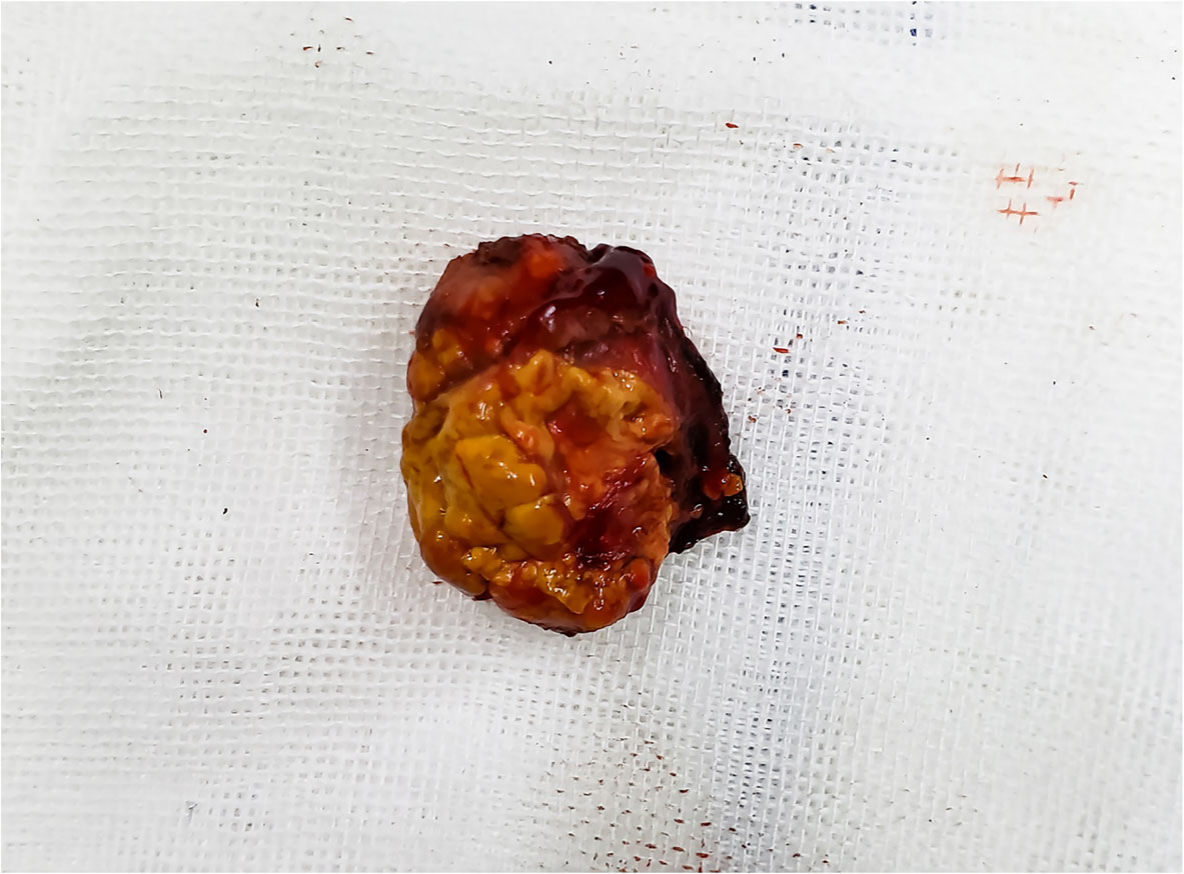
The surgical specimen size was approximately 2.1 cm × 2.0 cm × 1.8 cm

**Fig. 5:**
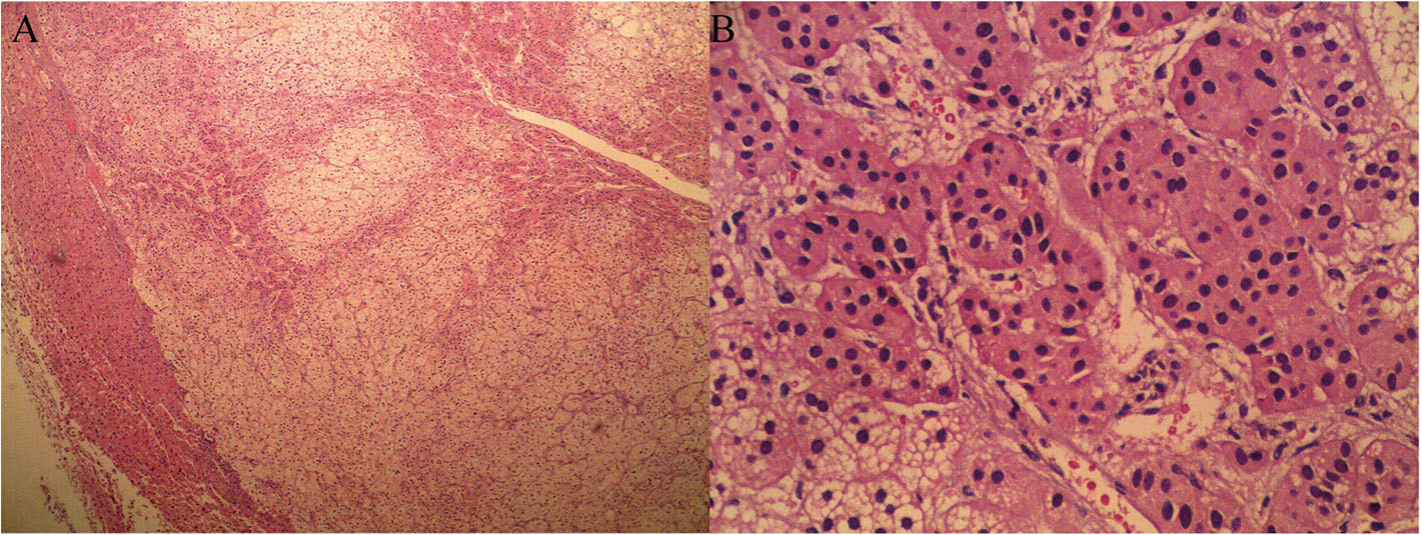
The tumor had clear boundaries, enveloped with a thin fibroid membrane and surrounded with some liver tissues. The tumor was composed of different proportions of bright and dark cells of acidophilic cytoplasm. These cells were distributed in cable or chests, accompanied by abundant blood vessels and sinusoidal structures (organ-like structures). The cell nucleus was round or oval and presented slight atypia. Aberrant cell nuclei were observed, and mitosis was rare (A. HE × 50; B, HE× 400)

**Fig. 6:**
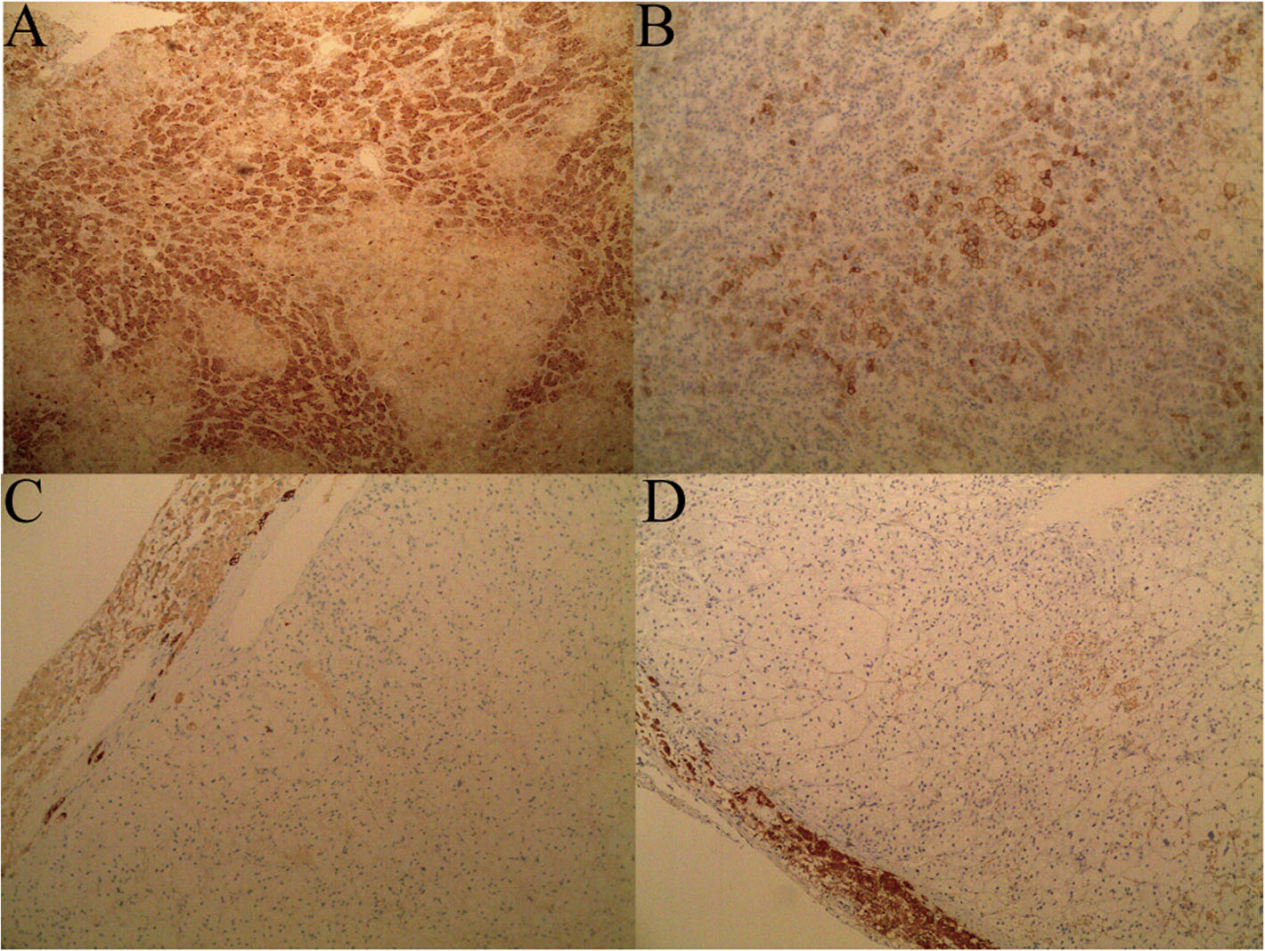
**a**. Tumor cells were positive for inhibin-α; **b**. Tumor cells were positive for synaptophysin; **c.** Tumor cells were negative for CK; **d**. Tumor cells were negative for hepatocytes

The patient was diagnosed with “primary hepatic carcinoma” before hospitalization. Routine blood examination, biochemical tests, and analyses of alpha fetoprotein (AFP), carcinoembryonic antigen (CEA), cancer antigen 125 (CA125) and cancer antigen 19–9 (CA19–9), three systems of hepatitis B, hepatitis C antibody, HIV, RPR, and coagulation function were conducted. The results showed no obvious anomalies. According to B-ultrasound reexamination, a 2.1 cm × 1.6 cm echo node was found on the right lobe of the liver, and an even internal echo and clear boundary was detected (Fig. 1). Enhanced CT of the abdomen showed an irregularly enhanced node at the seventh section of the arterial phase. The node contained edges enhanced in a circular manner and few fat dense particles. The enhancement was evident during the venous portal and delayed stages. The focus size was 2.2 cm × 1.8 cm, showing clear boundaries (Fig. 2). Enhanced magnetic resonance imaging (MRI) revealed an abnormal signal of the nodule (20 mm × 18 mm with clear boundary) at the S7 section of the liver. T1WI presented equisignals, and the antiphase was considerably lower than the in-phase. T2WI and T2WI + FS presented slightly higher signals, diffusion-weighted imaging (DWI) presented a high signal, and the apparent diffusion coefficient (ADC) presented a slightly lower signal. These signals were considerably enhanced during enhanced arterial phase scanning but disappeared during the venous portal and delayed stages. All signals detected were located lower than the liver parenchyma (Fig. 3). These results led to the diagnosis of hepatocellular carcinoma, and hepatectomy was planned. Under general anesthesia, the patient underwent open hepatectomy. Resection was performed under the guidance of intraoperative ultrasonography. It took about 180 min and caused 50 ml blood loss. The surgical specimen size was approximately 2.1 cm × 2.0 cm × 1.8 cm (Fig. 4). Hematoxylin/eosin (HE) staining was conducted in accordance with the pathological examination results. The tumor had clear boundaries, enveloped with a thin fibroid membrane, and was surrounded with some liver tissues. The tumor was composed of different proportions of bright and dark cells of acidophilic cytoplasm. These cells were distributed in cable or chests, accompanied by abundant blood vessels and sinusoidal structures (organ-like structures). The cell nuclei were round or oval and presented slight atypia. Aberrant cell nuclei were observed, and mitosis was rare (Fig. 5). Immunohistochemical examination showed the following results: inhibin-α (+) (Fig. 6.a), synaptophysin (+) (Fig. 6.b), melan-A (−), CK (−) (Fig. 6.c), cyclin D1 (−), chromogranin A (−), P53 (−), and hepatocytes (−) (Fig. 6.d). The final pathological diagnosis was adrenal cortical adenoma. The patient was discharged from the hospital after the operation. B-ultrasonication was performed 6 months after surgery and 1 year after surgery. The patient showed no relapse after 1 year of follow-up visits.

## Discussion and conclusions

Although hepatic adrenal ectopia is a common pathological state, cases where it causes adrenal tumors are rare. To date, only ten cases of hepatic adrenal tumors have been reported in English (Table 1), and all these cases were from Asia. The clinical manifestations, CT examination, pathological results, and immunohistochemical results of these cases were analyzed. Among the ten patients, only one was diagnosed with functional adenoma, which was recognized as aldosteronoma [7]. The nine remaining patients did not develop functional tumors. Nine patients underwent excision for highly suspected liver cancer, and only one patient underwent radiofrequency ablation treatment because of refusal to undergo surgery. A clear diagnosis of hepatic adrenal tumor cannot be made according to the imaging data and clinical manifestations. Hepatic adrenal tumors are often diagnosed as liver cancer or metastatic liver tumors. Accurate diagnosis of hepatic adrenal tumors are vital to avoid unnecessary liver excision.

**Table 1:** literature case reports summary

Year of publication	Country	Year	Sex	Diameter (cm)	Chief complaint	Diagnosis	Therapy	Immunohistochemistry
2017 [2]	Korea	56	F	3.4	“indigestion”	Nonfunctional adrenal adenoma	hepatectomy	alpha-inhibin(+) melan-A(+) synaptophysin(+)
2017 [2]	Korea	75	M	6.0	“cholecystolithiasis”	Nonfunctional adrenal adenoma	hepatectomy	alphainhibin(+) melan-A(+)
2017 [2]	Korea	64	F	2.2	“urinary tract infection”	Nonfunctional adrenal adenoma	hepatectomy	alpha-inhibin(+) melan-A(+) synaptophysin(+) glypican-3(+)
2016 [7]	Korea	45	F	1.4	“Hypertension”	Aldosterone producing adenoma	Radiofrequency Ablation	not available
2009 [8]	Korea	45	F	3.5	“Leiomyoma of uterus”	Nonfunctional adrenal adenoma	hepatectomy	not available
2013 [9]	Japan	60	M	3.0	“Diabetes”	Nonfunctional adrenal adenoma	hepatectomy	not available
2007 [10]	Korea	66	F	1.5	“rectal cancer”	Nonfunctional adrenal adenoma	hepatectomy	not available
2010 [11]	Korea	45	M	2.7	“heavy alcoholism”	Nonfunctional adrenal adenoma	tumorectomy	not available
2019 [12]	Korea	59	F	1.5	“Hepatic nodules”	Nonfunctional adrenal adenoma	laparoscopic surgical resection	not available
2019 [12]	Korea	75	F	2.5	“rectal nonmucinousadenocarcinoma”	Nonfunctional adrenal adenoma	ultrasound-guided core needle biopsy	alpha-inhibin(+) melan-A(+)

Hepatic adrenal adenoma has no unique clinical symptoms, except the presence of malignant or functional lesions. Patients are often diagnosed by imaging examination. However, no unique changes are detected in the imaging diagnosis of hepatic adrenal adenoma [13]. Adrenal tumors have rich lipid content and can be easily detected by B ultrasonic examination, MRI, and ordinary CT [14]. Hepatic adrenal tumors develop by fusion of adrenal liver tissues and might be misdiagnosed as metastatic or malignant tumors. Park conducted an imaging analysis of hepatic adrenal adenoma and concluded that it might be diagnosed if the plain CT scan value of the lesion is less than or equal to 10 HU and when the lesion is continuous with the right adrenal gland on multiplanar reconstruction images [8, 15]. In our reported case, the average ordinary CT value of the tumor was 8 HU (Fig. 2.F). Abdominal CT showed ambiguous boundaries between the right liver node and the lateral branch on the right adrenal gland (Fig. 2.E). Therefore, hepatic adrenal adenoma was highly suspected based on these imaging manifestations. Kawasaki believed adrenocortical SPECT/CT should be taken into consideration to diagnose hepatic adrenal rest [16]. There was obvious uptake in the liver lesion and faint uptake in the adrenal glands. Similarly, based on similar pathological basis, we believe that using of adrenocortical SPECT/CT may be helpful for the diagnosis of hepatic adrenal adenoma. Despite the rapid development of imaging, core-needle biopsy still remains the gold standard for diagnosing liver diseases [17]. In our hospital, we often use ultrasound-guided 18-gauge core-needle biopsy. In this case, percutaneous liver core-needle biopsy could be a suitable diagnostic method before the surgery.

The final diagnosis of hepatic adrenal adenoma requires pathological examination. Hepatic adrenal adenoma can be successfully diagnosed in terms of its morphology and the existence of residual normal heterotopic adrenal cortical tissues. Immunohistochemical examination is conducive to distinguishing hepatocellular carcinoma from metastatic cancer. In adrenocortical adenoma, synaptophysin, inhibin-α, and melan-A might be positive, and hepatocytes, which are generally related to liver cancer, are negative. In the present case, inhibin-α (+), synaptophysin (+), and hepatocytes (−) were detected on pathological immunohistochemical examination. However, few relevant cases have been reported, and a unified diagnosis standard has not been established. In particular, collecting relevant clinical information by fine-needle aspiration biopsy is necessary upon suspicion of adrenal tumors. Attention should be paid to the patient’s history of hypertension, chronic liver disease, and phymatosis. Examination results, such as AFP and CEA results, should be considered, and appropriate immunohistochemical staining should be applied for final accurate diagnosis.

According to this study, the possibility of hepatic adrenal adenoma should be considered for patients diagnosed with hepatic carcinoma by imaging examination. Diagnosis should be made by combining data on disease history, blood examination, imaging characteristics, and biopsy if necessary. The diagnosis of hepatic adrenal adenoma should be ruled out to avoid unnecessary operation. Analysis of a large number of patients is required to establish standards for the diagnosis of hepatic adrenal adenoma. It should be pointed out that hepatic adrenal adenoma has malignant potential, it represent conditions requiring therapeutic intervention (e.g. larger than 4 cm, the lesion enlarges by more than 20% after 6–12 months). We suggest performing hepatectomy through a laparoscopic access. However, laparoscopic surgery has higher requirements for the surgeons.

## Data Availability

All data produced and obtained is available within the manuscript.

## Abbreviations

CT: Computed tomography
MRI: Magnatic resonance imaging
AHA: Adrenohepatic adhesion
AHF: Adrenohepatic fusion
CEA: Carcinoembryonic antigen
AFP: Alpha fetoprotein
Hu: Hounsfield unit
CA 125: Cancer antigen 125
CA 199: Cancer antigen 199
CK: Cytokeratin
HE: Hematoxylin and eosin
Hu: Hounsfield unit
RPR: Rapid plasma reagin test
DWI: Diffusion weighted imaging
ADC: Apparent diffusion coefficient
